# Causal relationship between gut microbiota, blood metabolites and autism spectrum disorder: a Mendelian randomization study

**DOI:** 10.1098/rsos.250158

**Published:** 2025-05-21

**Authors:** Jigan Wang, Hui-Hong Dou, Qiong-You Liang

**Affiliations:** ^1^ Maternal and Child Health Hospital of Guangxi Zhuang Autonomous Region, Nanning, People’s Republic of China; ^2^ Department of Pediatrics, Guangxi Clinical Research Center for Pediatric Diseases, Nanning, People’s Republic of China

**Keywords:** ASD, gut microbiota, blood metabolite, MR, autism spectrum disorder

## Abstract

This study explored the causal relationships between gut microbiota, blood metabolites and autism spectrum disorder (ASD) in children and assessed whether metabolites mediate the relationship between microbiota and ASD. Using Mendelian randomization (MR), causal links between gut microbiota, blood metabolites and ASD were analysed, alongside reverse MR to examine reverse causality. A two-step MR mediation analysis was used to assess metabolite mediation. The study identified 15 gut microbiota types significantly associated with ASD, with Marinilabiliaceae showing the strongest positive link (odds ratio (OR) = 5.206, 95% confidence interval (CI) = 1.2783–21.2017, *p* = 0.0213) and Poseidoniaceae the strongest negative association (OR = 0.1466, 95% CI = 0.0306–0.7035, *p* = 0.0164). Among 52 blood metabolites, 4-methylcatechol sulphate was positively associated with ASD risk (OR = 1.6776, 95% CI = 1.0482–2.6849, *p* = 0.0311), while the glucose-to-maltose ratio showed a negative relationship (OR = 0.6358). No significant reverse causal effects of ASD on microbiota or metabolites were found. Nine metabolites mediated the relationship between microbiota and ASD, with 1-methyl-5-imidazoleacetate showing the strongest negative mediation effect (mediating effect = −0.0862, mediation proportion = 12.30%). This study reveals complex causal pathways involving microbiota and metabolites in ASD, suggesting metabolites may mediate the microbiota–ASD relationship, offering insights into ASD mechanisms and potential interventions.

## 1. Introduction

Autism spectrum disorder (ASD) is a complex neurodevelopmental disorder characterized by difficulties in social communication, language development delays, narrow interests and repetitive behaviours [1–3]. In recent years, the prevalence of ASD has been increasing, with current global rates estimated at approximately 1–2% [4]. Despite extensive research into the genetic, environmental and neurobiological mechanisms underlying ASD, its exact aetiology and pathophysiology remain unclear [5]. Recently, researchers have focused more on the potential role of gut microbiota in ASD development, as it has been identified as a significant factor associated with various neuropsychiatric disorders, with a particularly distinct role in ASD [6,7].

The *gut–brain axis* theory provides a biological framework for understanding the connection between the nervous system and gut microbiota. Studies suggest that gut microbiota may influence central nervous system function and behavioural outcomes through various pathways, such as metabolite release, immune regulation, neurotransmitter production and vagus nerve signalling [8]. Observational studies have reported that individuals with ASD often exhibit reduced gut microbiota diversity and abnormal proportions of specific microbial groups [9]. However, due to confounding factors, observational studies struggle to establish a clear causal relationship between gut microbiota and ASD. Meanwhile, blood metabolites, as intermediate products of host–gut microbiota interactions, not only reflect gut microbiota’s metabolic activity but may also directly or indirectly influence brain function. Changes in certain metabolite levels have been closely linked to ASD behavioural traits and cognitive functions, suggesting that metabolites could play a crucial mediating role between gut microbiota and ASD [10].

Mendelian randomization (MR) analysis is a statistical method that uses genetic variations as instrumental variables to study causal relationships, overcoming confounding factors and reverse causality issues inherent in traditional observational studies. Compared to conventional correlation analysis, MR analysis can more effectively reveal the causal pathways between gut microbiota, blood metabolites and ASD. Additionally, two-step MR mediation analysis can further uncover the potential mediating role of blood metabolites between gut microbiota and ASD [11].

Based on this background, this study systematically applies MR and mediation analyses to elucidate the causal effects of gut microbiota and blood metabolites on ASD and to identify potential mediating mechanisms. This not only deepens our understanding of ASD aetiology but also provides new insights and theoretical foundations for potential future intervention strategies and treatments.

## 2. Material and methods study design

**Figure 1.**
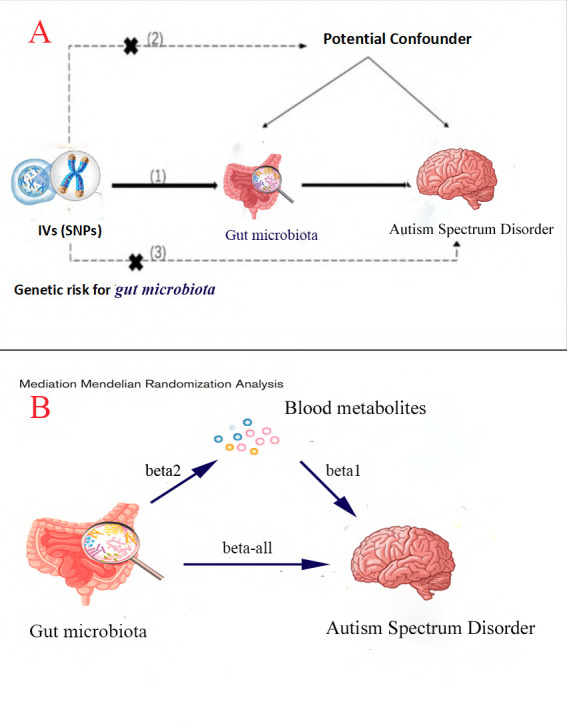
(A) Schematic diagram of the two-sample MR analysis on the association between exposure and outcome. (B) Mediation analysis design diagram.

We first utilized a two-sample MR analysis to evaluate the causal relationships between gut microbiota, plasma metabolites and ASD. The procedures followed the latest STROBE-MR guidelines [12] and were conducted under the following three key assumptions: (i) The instrumental variable must show a significant association with the exposure. (ii) The instrumental variable should be independent of any confounding factors. (iii) The instrumental variable should influence the outcome (ASD) solely through its effect on the exposure (gut microbiota) [13] (refer to figure 1A for details).

### 2.1. Mediation analysis

We employed a two-step MR approach to estimate the indirect effects of plasma metabolites as mediators of the causal influence of gut microbiota on ASD. Two-step MR involves calculating the following two MR estimates: (i) The causal effect of the mediator on the outcome (beta 1). (ii) The causal effect of the exposure on the mediator (beta 2).

The indirect effect of the mediator can be calculated as the product of these two estimates (beta 1 × beta 2) [14]. Here, beta-all represents the total effect of the exposure on the outcome, as determined by the two-sample MR analysis. The proportion of mediation is calculated by dividing the indirect effect by the total effect: beta 1 × beta 2/beta-all [11,14] (figure 1B).

### 2.2. Data sources

— *Gut Microbiota Data*: The data were retrieved from the GWAS (Genome-wide association study) database (https://www.ebi.ac.uk/gwas/) with study IDs ranging from GCST90032172 to GCST90032644, which include information on 2801 microbial taxa and 7 967 866 human genetic variants. This dataset represents a European population of 5959 individuals from the FR02 cohort, covering a wide taxonomic range: 11 phyla, 19 classes, 24 orders, 62 families, 146 genera and 209 species [15].
— *Plasma Metabolite and Juvenile Idiopathic Arthritis GWAS Data*: Retrieved from the GWAS Catalogue (GCST90199621–GCST90204603, GCST90010715), available at http://ftp.ebi.ac.uk/pub/databases/gwas/summary_statistics/. Plasma metabolite data involve 8299 individuals of European ancestry, encompassing 1091 blood metabolites and 309 metabolite ratios, with around 150 000 single nucleotide polymorphisms (SNPs) [16].
— *ASD Data*: Obtained from the Finnish database (https://r10.finngen.fi/), including 646 ASD cases and 302 819 controls.

### 2.3. Single nucleotide polymorphism selection for gut microbiota and blood metabolites

The screening criteria for gut microbiota and plasma metabolite SNPs were initially set at *p* < 5 × 10^−8^. However, this threshold only identified a small number of SNPs. To avoid inaccurate results due to a small sample size, the *p*-value threshold was adjusted to 5 × 10^−5^ [17]. Subsequently, a linkage disequilibrium clustering method was applied to exclude unnecessary SNPs (*r*^2^ < 0.001, window size = 10 000 kb). For palindromic SNPs, the forward strand was determined based on allele frequency. The selected IVs (Instrumental Variables) had *F*-values >10, ensuring that causal estimates were not influenced by weak instrument bias [18].

### 2.4. Statistical analysis

The two-sample MR analysis was performed using the ‘Two Sample MR’ package in R (v. 4.3.3). The inverse variance weighting method was employed to assess the pairwise causal relationships between the gut microbiota, metabolites and ASD. Only gut microbiota and plasma metabolites with a *p*‐value <0.05 and no evidence of heterogeneity or pleiotropy were included in the analysis. Heterogeneity of the MR estimates was evaluated using Cochrane’s *Q* test [19]. To examine potential horizontal pleiotropy, we used MR-PRESSO and MR–Egger regression, with *p* < 0.05 considered as significant [20].

## 3. Results

### 3.1. Selection of instrumental variables

When the gut microbiota was used as the exposure factor, a total of 269 SNPs strongly associated with 15 gut microbial taxa were included in the analysis, with *F*-values ranging from 19.65 to 20.88 (electronic supplementary material, table S1). When plasma metabolites were used as the exposure factor, a total of 1297 SNPs strongly associated with 52 metabolites were included in the analysis, with *F*-values ranging from 19.81 to 98.78 (electronic supplementary material, table S2).

### 3.2. Causal relationship between gut microbiota and autism spectrum disorder

In the genetic analysis of gut microbiota related to ASD, we identified 15 types of gut microbiota with a causal relationship with ASD. Among them, Agathobacter sp000434275, CAG-194 sp002441865, Cyanobacteria, Gordonibacter, Haemophilus D sp001679485, Rubneribacter sp002159915, Marinilabiliaceae, Pararhizobium and Turicibacter were positively associated with ASD risk, with Marinilabiliaceae showing the strongest positive correlation (odds ratio (OR) = 5.206, 95% confidence interval (CI) = 1.2783–21.2017, *p* = 0.0213).

**Figure 2.**
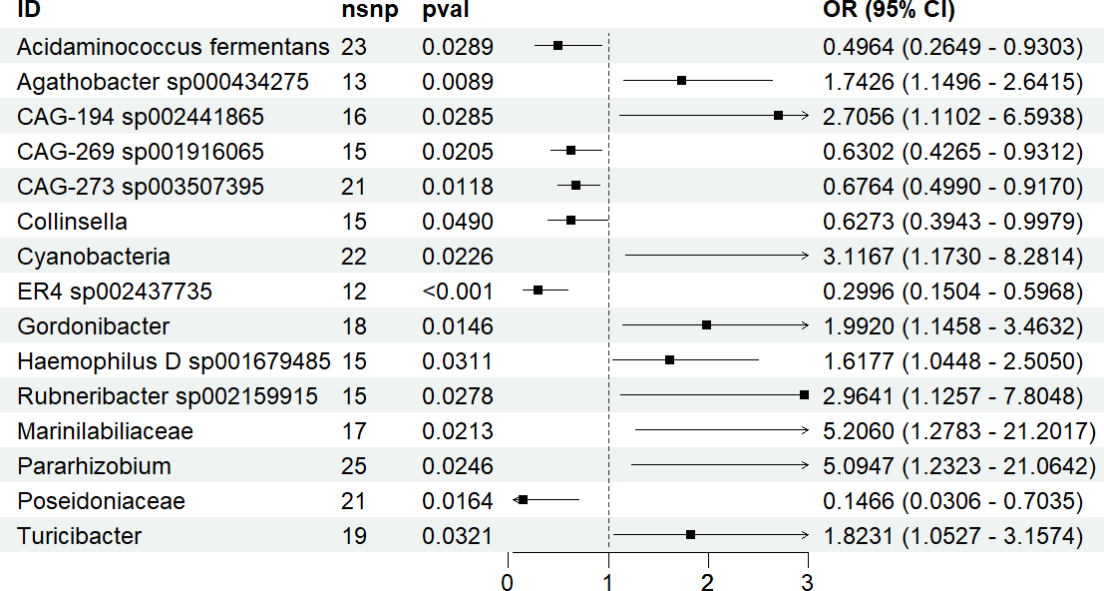
Results of MR analysis of gut microbiota and ASD.

On the other hand, Acidaminococcus fermentans, CAG-269 sp001916065, CAG-273 sp003507395, Collinsella, ER4 sp002437735 and Poseidoniaceae were negatively associated with ASD risk, with Poseidoniaceae showing the strongest negative correlation (OR = 0.1466, 95% CI = 0.0306–0.7035, *p* = 0.0164; figure 2). These results were validated through sensitivity analyses, confirming no heterogeneity or horizontal pleiotropy (electronic supplementary material, table S3).

### 3.3. Causal relationship between blood metabolites and autism spectrum disorder

**Figure 3.**
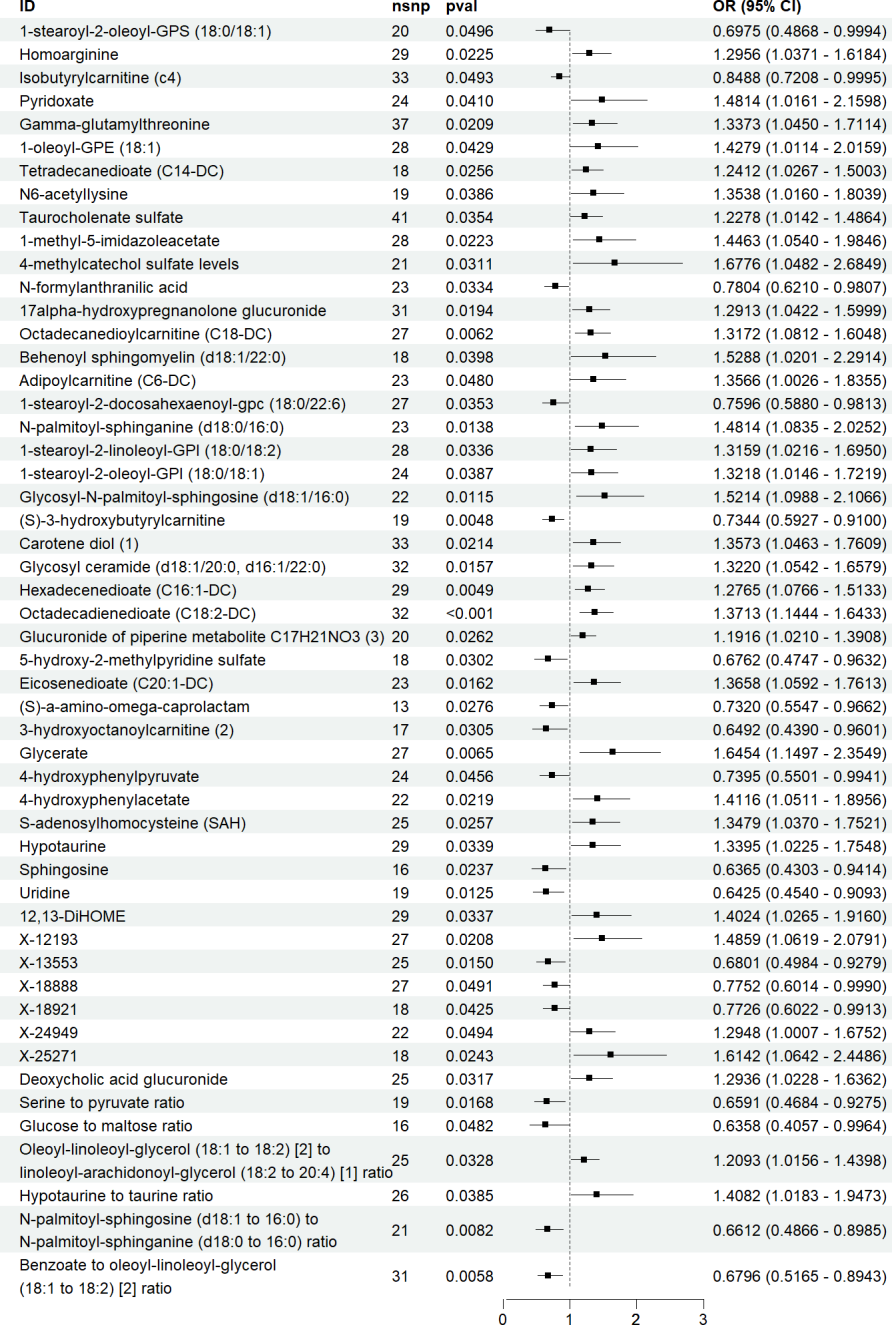
Results of MR analysis of blood metabolites and ASD.

We identified 52 blood metabolites with a causal relationship with ASD. Of these, 34 metabolites showed a positive correlation with ASD risk, with 4-methylcatechol sulphate being the most significant (OR = 1.6776, 95% CI = 1.0482–2.6849, *p* = 0.0311). Meanwhile, 18 metabolites were negatively associated with ASD risk, with glucose-to-maltose ratio exhibiting the strongest negative correlation (OR = 0.6358, 95% CI = 0.4057–0.9964, *p* = 0.0482; figure 3). Sensitivity analyses confirmed no heterogeneity or horizontal pleiotropy in these results (electronic supplementary material, table S4).

### 3.4. Causal relationship between gut microbiota and blood metabolites

**Figure 4.**
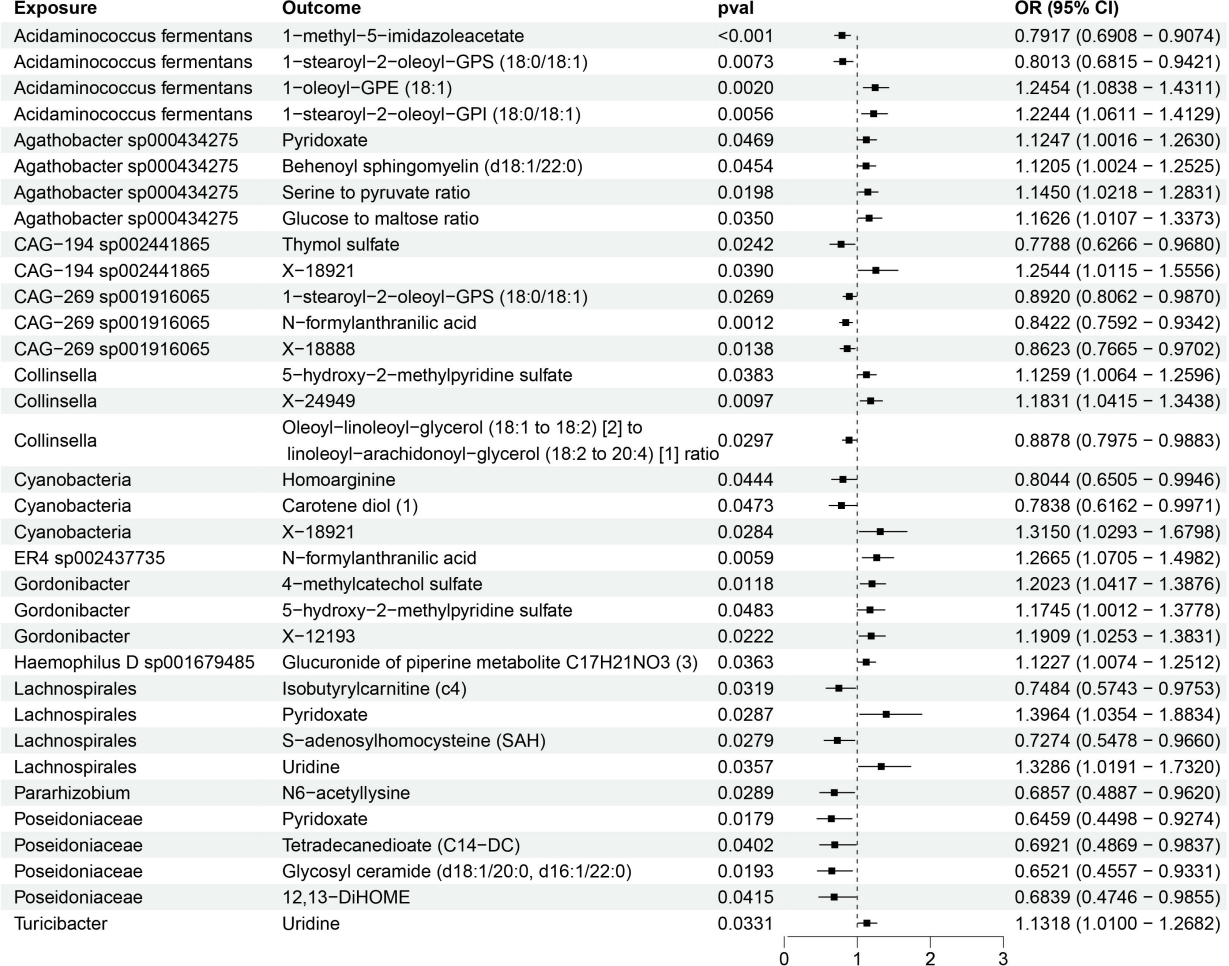
Results of MR analysis of gut microbiota and blood metabolites.

Among the previously identified gut microbiota and plasma metabolites associated with ASD, we found 15 gut microbiota types and 52 blood metabolites related to ASD. We performed MR analysis using these 15 types of gut microbiota as exposure factors and 52 blood metabolites as outcome indicators, resulting in 34 positive causal associations between gut microbiota and blood metabolites (figure 4).

### 3.5. Reverse Mendelian randomization analysis

To assess the potential reverse causal effects of ASD on these 15 gut microbiota and 52 blood metabolites, we conducted a reverse MR analysis. The results showed that ASD had no significant reverse causal effects on the gut microbiota (electronic supplementary material, figure S1) or blood metabolites (electronic supplementary material, figure S2). Among the 34 positive causal relationships identified between the gut microbiota and blood metabolites, reverse MR analysis was performed to confirm the absence of reverse causality (electronic supplementary material, figure S3).

### 3.6. Mediation analysis

To identify potential mediating blood metabolites between gut microbiota and ASD, we performed a two-step MR mediation analysis on the 34 positive results. The analysis revealed that nine metabolites mediated the relationship between five gut microbiota and ASD, resulting in nine positive mediation effects. Among these, three were positive associations, and six were negative associations. The strongest mediation effect was from 1-methyl-5-imidazoleacetate, mediating the effect of Acidaminococcus fermentans on ASD (mediation effect = −0.0862, mediation proportion = 12.30%, 95% CI = 3.49–21.12%). The second strongest mediation effect was from pyridoxate, mediating the effect of Lachnospirales on ASD (mediation effect = 0.1312, mediation proportion = 12.08%, 95% CI = 3.69–20.47% (see table 1).

**Table 1.** The mediating effect of blood metabolites on ASD through the gut microbiota

exposeure	intermediate factor（blood metabolites）	outcome	mediating effect	direct effect	mediating effect ratio（95% CI）
Acidaminococcus fermentans	1-methyl−5-imidazoleacetate	ASD	−0.0862	−0.6141	12.30%（3.49%–21.12%）
ER4 sp002437735	N-formylanthranilic acid	ASD	−0.0586	−1.1467	4.86%（1.93%–7.79%）
Haemophilus D sp001679485	Glucuronide of piperine metabolite C17H21NO3 (3)	ASD	0.0203	0.4607	4.22%（1.05%–7.38%）
Lachnospirales	Isobutyrylcarnitine (c4)	ASD	0.0475	1.0391	4.37%（0.55%–8.19%）
Lachnospirales	Pyridoxate	ASD	0.1312	0.9553	12.078%（3.69%–20.47%）
Poseidoniaceae	Pyridoxate	ASD	−0.1718	−1.7482	8.95%（3.19%–14.71%）
Poseidoniaceae	Tetradecanedioate (C14-DC)	ASD	−0.0795	−1.8405	4.14%（0.53%–7.75%）
Poseidoniaceae	Glycosyl ceramide (d18:1/20:0, d16:1/22:0)	ASD	−0.1193	−1.8007	6.21%（1.81%–10.62%）
Poseidoniaceae	12,13-DiHOME	ASD	−0.1285	−1.7915	6.69%（2.06%–11.32%）

## 4. Discussion

This study systematically explored the causal relationships between gut microbiota, blood metabolites and ASD using MR and mediation analyses, uncovering potential mediating effects of certain metabolites between gut microbiota and ASD. The findings suggest that specific gut microbiota and blood metabolites have significant causal associations with ASD development and exert potential effects on ASD through metabolite mediation.

### 4.1. Causal association between gut microbiota and autism spectrum disorder and potential mechanisms gut microbiota’s causal role in autism spectrum disorder

The analysis identified 15 types of gut microbiota significantly associated with ASD, with Marinilabiliaceae showing the strongest positive association and Poseidoniaceae demonstrating a significant negative association. The underlying mechanisms may involve the following.

#### 4.1.1. Regulation of neurotransmitters by gut microbiota

Gut microbiota is closely linked to the synthesis and regulation of neurotransmitters. For instance, it can influence serotonin (5-HT) production via the tryptophan metabolic pathway, and 5-HT plays a critical role in regulating mood, social behaviour and cognitive functions [21]. In patients with ASD, gut microbiota may disrupt serotonin metabolism and transport, leading to neurotransmitter imbalances, which could partially explain social communication deficits and emotional regulation issues. Moreover, the synthesis and function of other neurotransmitters, like gamma-aminobutyric acid (GABA), may also be regulated by gut microbiota. Abnormalities in these neurotransmitters might be associated with the neuropathological features of ASD [22].

#### 4.1.2. Impact of gut microbiota metabolites on neurodevelopment

Metabolites produced by gut microbiota, such as short-chain fatty acids (SCFAs), bile acids and tryptophan derivatives, have been shown to cross the blood–brain barrier and affect neurodevelopment and neural function [23]. For example, propionate, a type of SCFA, is believed to affect neuronal signalling and synaptic plasticity by activating specific G-protein-coupled receptors [24]. Research has indicated that the abnormal levels of certain SCFAs in patients with ASD may lead to neurodevelopmental issues, such as excessive excitatory neurotransmission or reduced inhibitory signals, which could be linked to ASD behavioural traits [25].

#### 4.1.3. Gut–brain signalling mechanisms

The gut–brain axis, particularly the vagus nerve pathway, allows gut microbiota to directly influence central nervous system functions. The vagus nerve, which governs nearly the entire digestive system, represents one of the most direct and well-studied communication routes between the gut and the brain, providing a mechanism for gut microbiota to modulate brain activity [26]. Bacterial taxa (i.e. *Campylobacter jejuni*, *Lacticaseibacillus rhamnosus* JB-1 a.k.a. *Lactobacillus rhamnosus*/*reuteri* JB-1, *Limosilactobacillus reuteri* a.k.a. *Lactobacillus reuteri*) have been shown to affect brain, cognition and behaviour via vagal signalling, resulting in positive and negative outcomes [27,28].

#### 4.1.4. Microbial gene function influence

The genetic functions of gut microbiota may impact neuronal development and synaptic plasticity by encoding specific proteins or metabolic enzymes [29]. This offers a new perspective on the genetic and environmental factors of ASD, suggesting that modulating microbial gene function could be a potential strategy for future ASD interventions.

### 4.2. Causal association between blood metabolites and autism spectrum disorder

In the blood metabolite analysis, 52 metabolites were identified as significantly associated with ASD. Among these, 4-methylcatechol sulphate showed the strongest positive correlation with ASD risk, while the glucose-to-maltose ratio had the strongest negative correlation. The positive association of 4-methylcatechol sulphate with ASD suggests the potential role of metabolic dysregulation in ASD development, particularly in neurotransmitter synthesis and metabolic pathways. Meanwhile, the negative correlation of the glucose-to-maltose ratio may highlight the importance of energy metabolism regulation in ASD pathology, consistent with previous observations of abnormal energy metabolism in ASD patients [30,31].

## 5. Significance of mediation analysis

The two-step MR mediation analysis revealed significant mediating effects of eight metabolites between gut microbiota and ASD. These mediating metabolites not only serve as bridges between gut microbiota and brain function but may also regulate ASD’s pathological processes through multiple metabolic pathways.

Notably, 1-methyl-5-imidazoleacetate exhibited the strongest negative mediation effect between Acidaminococcus fermentans and ASD, 1-methyl-5-imidazoleacetate is a product of the histidine metabolism pathway and has been identified in some studies as one of the metabolites derived from gut microbial activity. The histidine metabolic pathway plays a crucial role in various physiological and pathological processes, including inflammatory responses and neurotransmitter regulation. It may indirectly influence neurotransmitter modulation by affecting histamine metabolism, thereby potentially contributing to the development or symptomatology of ASD. [32]. In contrast, pyridoxate showed a positive mediation effect between Lachnospirales and ASD, supporting the crucial role of vitamin B6 in neurotransmitter synthesis and neurodevelopment [33,34]. As a coenzyme in glutamate and GABA synthesis, changes in vitamin B6 levels can significantly affect the balance of inhibitory and excitatory signals in the nervous system [35].

The strength of the mediation effects varied significantly among different microbial taxa and metabolites. A stronger mediation effect, such as 1-methyl-5-imidazoleacetate mediating 12.30% of the causal pathway between Acidaminococcus fermentans and ASD, indicates a more crucial role for the metabolite in linking gut microbiota to ASD. Its effect size is −0.0862. Although this effect size is relatively small, it may still hold potential significance in both biological and clinical contexts. The intensity of these mediation effects may reflect the multiple biological functions of metabolites in ASD pathogenesis, including neurotransmitter synthesis regulation, cellular energy metabolism and oxidative stress response. Additionally, the positive or negative effects of different mediating metabolites suggest distinct pathways through which they influence ASD risk, providing a new dimension for understanding the complex impact of gut microbiota on ASD.

As products of host–microbiome interactions, metabolites exhibit multifunctionality and complex mechanisms of action, involving neuroinflammation, oxidative stress and the interplay between the gut microbiota and the central nervous system. In addition to serving as mediating signals, they may also regulate the composition and function of the gut microbiota through feedback mechanisms. For instance, SCFAs not only affect neurotransmitter metabolism but can also alter the gut microbiome’s ecosystem by regulating gut pH [36,37]. This bidirectional regulatory mechanism increases the complexity of gut microbiota’s association with ASD and highlights the central role of metabolites in gut–brain communication.

## 6. Study limitations

Although this study provides strong evidence for the mediating role of metabolites, several limitations should be acknowledged. First, the lack of dynamic measurements of metabolites: single-timepoint measurements may not fully capture the dynamic changes in metabolite levels. Since ASD is a long-term progressive disorder, metabolic profiles may vary significantly across developmental stages. Second, the diversity of causal pathways: although mediation analysis identified a mediating role of metabolites between the gut microbiota and ASD, the actual causal pathways may be more complex. Metabolites might influence the development of ASD through multiple mechanisms that are not fully captured by mediation analysis. Additionally, this study was based on GWAS data from a European population, and the generalizability of the findings requires further validation. Future studies should include diverse populations from different ethnic and regional backgrounds. Third, since the plasma metabolites and gut microbiota analysed in this study are limited, not all metabolites and microbial taxa that may influence autism have been covered. Therefore, the bidirectional effects of specific microbial communities cannot be completely ruled out. Future studies could integrate larger-scale metabolomic and microbiome data to draw more comprehensive conclusions. Lastly, although MR effectively reduces confounding and reverse causality, environmental confounders affecting the microbiome and metabolites (such as diet and antibiotic use) cannot be entirely ruled out.

## 7. Conclusion and future directions

This study uncovers the complex causal pathways between gut microbiota, metabolites and ASD, quantifying the mediating role of metabolites within this causal chain. Potential interventions targeting specific gut microbiota, such as probiotics or dietary fibres, could offer new strategies for ASD prevention and treatment. The mediation effects of metabolites not only enhance our understanding of the gut–brain axis but also provide new insights into the biological mechanisms of ASD and potential intervention strategies.

Future research should focus on exploring the dynamic changes of metabolites and their impact at different developmental stages. Additionally, integrating multi-omics data (e.g. genomics, transcriptomics, metabolomics and microbiomics) to further analyse the interactions between gut microbiota, metabolites and ASD could facilitate the development of novel biomarkers and targeted intervention strategies.

## Data Availability

All data are sourced from the free Finngen database (https://www.finngen.fi/en) and IEU OpenGWAS (https://gwas.mrcieu.ac.uk/) databases. Supplementary material is available online [38].
